# Enhanced independent discriminative performance of elevated lipoprotein(a) for cardiovascular outcomes in patients with diabetes: a comparative analysis of optimal cutoff values

**DOI:** 10.3389/fendo.2026.1795181

**Published:** 2026-04-21

**Authors:** Song Wen, Yanju He, Xiucai Li, Zhimin Xu, Dan Liu, Jiyu Li, Ligang Zhou

**Affiliations:** 1 Department of Endocrinology, Shanghai Pudong Hospital, Fudan University, Pudong Medical Center, Shanghai, China; 2 Department of Rheumatology, Shanghai Pudong Hospital, Fudan University, Pudong Medical Center, Shanghai, China; 3 Department of Surgery, Shanghai Pudong Hospital, Fudan University, Pudong Medical Center, Shanghai, China

**Keywords:** cardiovascular diseases (CVD), cutoff, diabetes mellitus, Lp(a), risk stratification

## Abstract

**Objective:**

This study aimed to evaluate the independent discriminative performance of elevated Lipoprotein(a) [Lp(a)] in identifying prevalent cardiovascular disease (CVD), specifically in patients with Diabetes Mellitus (DM), and to determine the optimal cutoff values for identifying CVD in the DM population.

**Methods:**

A stratified analysis was conducted across general, DM, and non-DM patient groups. Correlation analysis was employed to assess the association between elevated Lp(a) levels and metabolic factors in different age groups. Furthermore, binary regression was used to calculate combined risk scores. Receiver operating characteristic (ROC) curve analysis was utilized to determine the area under the curve (AUC) for the discriminative performance of Lp(a) alone, traditional parameters (excluding Lp(a)), and the combined model. This analysis identified the optimal cutoff values for each group.

**Results:**

Comparisons of Lp(a) variations showed that Lp(a)>300mg/L was associated with an increased prevalence of CVDs in general and non-diabetic patients, while it was insignificant in patients with DM, unless the cutoff was set as low as 70mg/L; Correlation analyses showed that, regardless of minor nuances between the general and DM groups, Lp(a) was significantly related to Low-density lipoprotein (LDL) and Apolipoprotein B (ApoB), but negatively related to Glycated Hemoglobin A1c (HbA1c), Triglycerides (TG), and free triiodothyronine (FT3); when stratified by age, no correlation was associated with Lp(a), but an association was found with CVD in the 65–75 age group, while Non-alcoholic fatty liver disease (NAFLD) prevalence was higher in the <65 age group across groups except for Lp(a)>70mg/L in the DM group; correlational analyses revealed that Lp(a) was positively related with CVD in the <65 age group compared to the general group. Regression analyses revealed that HbA1c and age significantly contributed to increased CVD in DM and that Lp(a) was determined by FT3 and albumin (Alb) in DM; ROC curves demonstrated that the combination of Lp(a) with traditional parameters significantly enhanced the AUC for CVD in DM.

**Conclusion:**

Elevated Lp(a) levels are significantly associated with CVD and demonstrate strong discriminative utility, particularly in patients with DM. These findings suggest that more stringent Lp(a) thresholds may be warranted in the clinical management of diabetic patients to better identify individuals at high risk for cardiovascular outcomes.

## Highlights

- The optimal discriminative threshold of Lp(a) for identifying prevalent CVD in diabetic patients was determined to be 70 mg/L.
- This 70 mg/L cutoff is approximately four-fold lower than the optimal threshold identified for the non-DM population (~270–300 mg/L), suggesting a heightened sensitivity to Lp(a) levels in the diabetic milieu.
- The integration of Lp(a) into established discriminative models significantly augmented the AUC for identifying CVD, confirming its role as a key independent factor associated with residual cardiovascular burden specific to the DM environment.
- Lp(a) showed an unexpected negative correlation with HbA1c and Triglycerides, underscoring the complex, non-linear, and independent association of Lp(a) with CVD in the context of DM.

## 1 Introduction

Cardiovascular disease (CVD) remains the leading cause of morbidity and mortality worldwide (1), with Diabetes Mellitus (DM) acting as a powerful and pervasive accelerator of atherosclerotic risk (2). DM fundamentally alters the vascular environment, leading to chronic inflammation, endothelial dysfunction, and a heightened pro-thrombotic state (3). While aggressive control of traditional risk factors, particularly low-density lipoprotein cholesterol (LDL-C), is standard care, a substantial residual risk persists in patients with DM, driving the need to identify and target non-traditional pathogenic lipoproteins (4). Among these, Lp(a), a unique LDL-like particle characterized by the highly polymorphic apolipoprotein(a) moiety (5), has emerged as a genetically determined, causal, and independent risk factor for CVD (6). However, the precise interaction between the hyperglycemic environment of DM and the atherogenic potential of Lp(a) remains to be defined, obscuring its optimal clinical application in this vulnerable population (7–9).

The clinical management of Lp(a) presents several challenges, primarily because its circulating concentrations are largely determined by genetics and are minimally affected by lifestyle interventions or standard lipid-lowering therapies (10, 11). Crucially, evidence suggests that the diabetic milieu may not only increase baseline risk but also fundamentally amplifies the atherogenicity of Lp(a) itself. Recent biochemical evidence suggests that the diabetic milieu accelerates the non-enzymatic glycation of the lysine-rich domains of both the apolipoprotein(a) and apolipoprotein B-100 components of Lp(a) (12, 13). This structural modification, particularly the formation of Advanced Glycation End-products (AGEs) on the Lp(a) particle, fundamentally enhances its atherogenicity (14, 15). Glycated Lp(a) exhibits a significantly higher binding affinity for the subendothelial proteoglycan matrix, leading to increased intimal retention and subsequent foam cell formation (16, 17); Moreover, this qualitative alteration impairs the normal lysine-binding function of Lp(a) to fibrin, which, while appearing to reduce competition with plasminogen, actually results in a dysfunctional, pro-thrombotic particle that is more resistant to clearance and more prone to oxidative modification within the vascular wall (18, 19). Furthermore, the interplay of Lp(a) with key metabolic markers and age-related risk profiles requires explicit analysis to determine the residual risk (20). Consequently, Lp(a) acts as a more potent risk factor in patients with diabetes, even at lower circulating concentrations.

In light of the demonstrated limitations of current guidelines for patients with DM, this study was specifically designed to investigate the independent discriminative utility of elevated Lp(a) levels for prevalent CVD exclusively within the DM cohort. By conducting a meticulous stratified analysis, correlation analyses, and ROC curve assessment across general, DM, and non-DM groups, we aimed to address two central questions: first, we aimed to determine whether the discriminative performance (AUC) of a classification model is significantly enhanced by adding Lp(a) in DM patients; and second, we aimed to identify the optimal cutoff value for Lp(a) that effectively identifies the presence of CVD in this group. These findings are intended to advocate for the adoption of more stringent, individualized Lp(a) thresholds in the clinical management of DM to facilitate a more precise identification and targeted intervention of associated cardiovascular burden.

## 2 Materials and methods

### 2.1 Study design, setting, and ethics

This study was conducted as a retrospective, cross-sectional analysis utilizing de-identified patient data collected from the electronic health records (EHRs) of Shanghai Pudong Hospital between March 2023 and October 2025. The total study cohort comprised 183 adult patients who had complete records of lipid panels, metabolic markers, and documented cardiovascular disease (CVD) status. Following a rigorous screening for data completeness, 14 patients were excluded due to insufficient treatment history details, resulting in a final analytic sample of 169 patients. The patient population was stratified into three primary groups for comparative analysis: the General Group (n=169), the DM Group (n=126), and the Non-Diabetes Mellitus Group (n=43). Lp(a) Stratification: Among the total cohort (N = 183), 101 patients were identified as having significantly elevated Lp(a) levels, defined as Lp(a) > 300 mg/L. The remaining patients formed the lower Lp(a) group. Subsequent analyses, particularly in the DM cohort, further explored the lower risk threshold of Lp(a)> 70mg/L.

### 2.2 Study population and definitions

#### 2.2.1 Patient enrollment and group stratification

The initial cohort included all adult patients who underwent a comprehensive health checkup or admission and had complete records for a lipid panel, metabolic markers, and a documented history of CVD status. The total population was stratified into three primary groups based on their diabetic status: 1) General Group (All eligible patients). 2) Diabetes Mellitus (DM) Group: Patients who met the diagnostic criteria for DM. 3) Non-Diabetes Mellitus (non-DM) Group: Patients who were excluded from the DM criteria.

#### 2.2.2 Disease definitions

Diabetes Mellitus (DM): Defined as a previous clinical diagnosis of DM, a self-reported history of diabetes, active use of insulin or oral antidiabetic medications, or World Health Organization (WHO) diagnostic standards. CVD: Defined as a documented history of major atherosclerotic events, including myocardial infarction (MI), ischemic or hemorrhagic stroke, coronary revascularization (Percutaneous Coronary Intervention (PCI) or Coronary Artery Bypass Graft (CABG)), or established peripheral artery disease (PAD). NAFLD: Diagnosis was primarily confirmed by imaging studies (e.g., abdominal ultrasound or computerized tomography (CT) scan) or defined by established scoring models (e.g., Fatty Liver Index (FLI)) in the absence of excessive alcohol consumption. The characteristics of the included patients can be found in **Table 1**.

**Table 1:** Characteristics of the included patients with or without Diabetes Mellitus.

Characteristics	General (n=169)	DM (n=126)	Non-DM (n=43)	p value
Age (yrs)	65.00 (56.00, 71.00)	66.00 (56.00, 72.00)	61.00 (49.00,73.00)	0.24
BMI (kg/m^2^)	24.28 (22.6, 27.14)	23.88 (22.60, 27.04)	25.03 (22.04, 27.14)	0.960
**FPG (mmol/L)**	6.63(5.16, 9.38)	7.30 (5.63, 9.69)	4.97 (3.54,5.45)	**<0.0001**
**HbA1c %**	8.20 (6.50, 10.7)	9.20 (7.60, 11.20)	6.00 (5.70, 6.20)	**<0.0001**
Lipids				
**Lp(a) (mg/L)**	217.00 (70, 487)	151.00 (67.00, 399.00)	458.00 (330.5, 670)	**<0.0001**
Total cholesterol (mmol/L)	4.44 (3.61, 5.22)	4.36 (3.61, 5.28)	4.50 (3.66, 4.79)	0.817
**Triglycerides (mmol/L)**	1.33 (0.91, 2.07)	1.57 (0.95, 2.24)	1.12 (0.77, 1.44)	0.039*
**HDL (mmol/L)**	1.11 (0.96, 1.35)	1.05 (0.93, 1.30)	1.19 (1.00,1.44)	0.026*
LDL (mmol/L)	2.33 ± 0.83	2.36 ± 0.84	2.21 ± 0.81	0.973
**ApoA1 (g/L)**	1.1 (1.03, 1.36)	1.08 (1.01, 1.28)	1.18 (1.075, 1.38)	0.025*
ApoB (g/L)	1.01 ± 0.27	1.03 ± 0.27	0.92 ± 0.25	0.274
ApoE (mg/L)	33.60 (26.5, 41.8)	33.80 (26.5, 41.5)	29.65 (26.3, 44.6)	0.814
Statin use				0.290
**yes**	84	66	18	
**no**	85	60	25	
Liver and kidney				
**Albumin (g/L)**	39.21 (36.9, 41.7)	38.63 (36.20, 41.20)	40.60 (38.20, 44.00)	0.018*
eGFR(ml/min*1.73m^2^)	91.91 (78.86, 101.77)	90.70 (73.36, 100.04)	95.48 (82.98, 106.17)	0.299
Uric acid (μmol/L)	306.95 ± 84.36	307.56 ± 82.81	304.58 ± 91.93	0.793
SCr (μmol/L)	69.32 (59.3, 81.8)	72.10 (59.20, 82.40)	64.85 (54.8, 73.2)	0.706
Thyroid function				
**FT3 (pmol/L)**	5.24 ± 0.72	5.16 ± 0.71	5.53 ± 0.69	0.009***
FT4 (pmol/L)	15.57 (14.17 17.22)	15.61 (14.39, 17.2200)	14.54 (14.07, 17.42)	0.541
TSH (mIU/L)	1.87(1.29, 3.05)	1.79 (1.37, 2.93)	2.46 (1.36, 3.43)	0.213

BMI, body mass index; FPG, fasting plasma glucose; HbA1c, hemoglobin A1c; Lp(a), lipoprotein a; HDL, high-density lipoprotein; LDL, low-density lipoprotein; ApoA, apolipoprotein A; eGFR, estimated glomerular filtration rate; SCr, serum creatinine; FT3, free triiodothyronine; FT4, free thyroxine; TSH, thyroid-stimulating hormone. The Mann-Whitney test (for two groups) was used whenever the data were non-normally distributed or presented as medians. The independent samples t-test or One-way ANOVA was only used for strictly normally distributed data.The *: p<0.05; ***: p<0.001.The Bold values <0.05 stands for statistical significance.

#### 2.2.3 Biochemical and anthropometric measurements

Metabolic and organic parameters were evaluated by collecting blood samples from patients upon admission. Serum blood glucose, pancreatic islet function, HbA1C, electrolytes, thyroid function, and hepatic and renal function are all included in these laboratory parameters. A full-automatic biochemical analyzer (ADVIA Chemistry XPT, SIEMENS, USA) was used to analyze all biochemical indicators, including fasting blood glucose, hepatic function, and kidney function indicators. Over the previous three months, glycemic control was determined by analyzing HbA1C using a TOSOH G8, Janpn analyzer. C-peptide and thyroid function indicators were processed using chemiluminescence methods with a fully automated chemiluminescence immunoassay analyzer (ADIVA Centaur XPT, SIEMENS, USA).

### 2.3 Statistical analysis

Statistical analyses were performed using SPSS, version 26.0 (IBM, Chicago, IL, USA) and Prism (GraphPad, version 10.0). Descriptive and Comparative Statistics: Continuous variables were tested for normal distribution using the Kolmogorov-Smirnov test. Normally distributed data are presented as mean ± standard deviation (SD), and non-normally distributed data as median (Interquartile Range, IQR). Categorical variables are presented as counts (n) and percentages (%). Differences between two groups were assessed using an independent samples t-test or a Mann-Whitney U-test, and differences among multiple groups were assessed using a One-way Analysis of Variance (ANOVA) or a Kruskal-Wallis test. CVD prevalence was compared across Lp(a) cutoff groups using the chi^2^ test. Correlation and Subgroup Analysis: the Spearman correlation coefficient was utilized for non-parametric assessment of the association between Lp(a) levels and metabolic markers. Subgroup analyses were conducted by stratifying the DM cohort based on age and Lp(a) status, and to explore context-specific risk relationships. Predictive Modeling and Risk Assessment: Binary Logistic Regression analysis was employed to identify the independent factors associated with CVD prevalence in the DM group. The results are reported as Odds Ratios (OR) with 95% Confidence Intervals (CI). The regression model included Lp(a) and other variables that showed significant associations in the univariate analysis. A separate regression analysis examined factors correlated with Lp(a) levels in the DM group. The discriminative performance of Lp(a) for CVD in the DM group was evaluated using the Receiver Operating Characteristic (ROC) curve analysis. The optimal cutoff value for Lp(a) in the DM group was determined by maximizing the Youden Index (Sensitivity + Specificity - 1). To ensure the stability of the logistic regression models and to minimize the risk of overfitting, internal validation was performed using bootstrapping with 1,000 resamples. This= provided bias-corrected 95% confidence intervals for the model coefficients. A two-tailed p-value of <0.05 was considered statistically significant for all tests.

## 3 Results

### 3.1 Comparison of different stratified Lp(a) levels in the prevalence of CVD cases among the general, DM, and non-DM groups.

**Figure 1:**
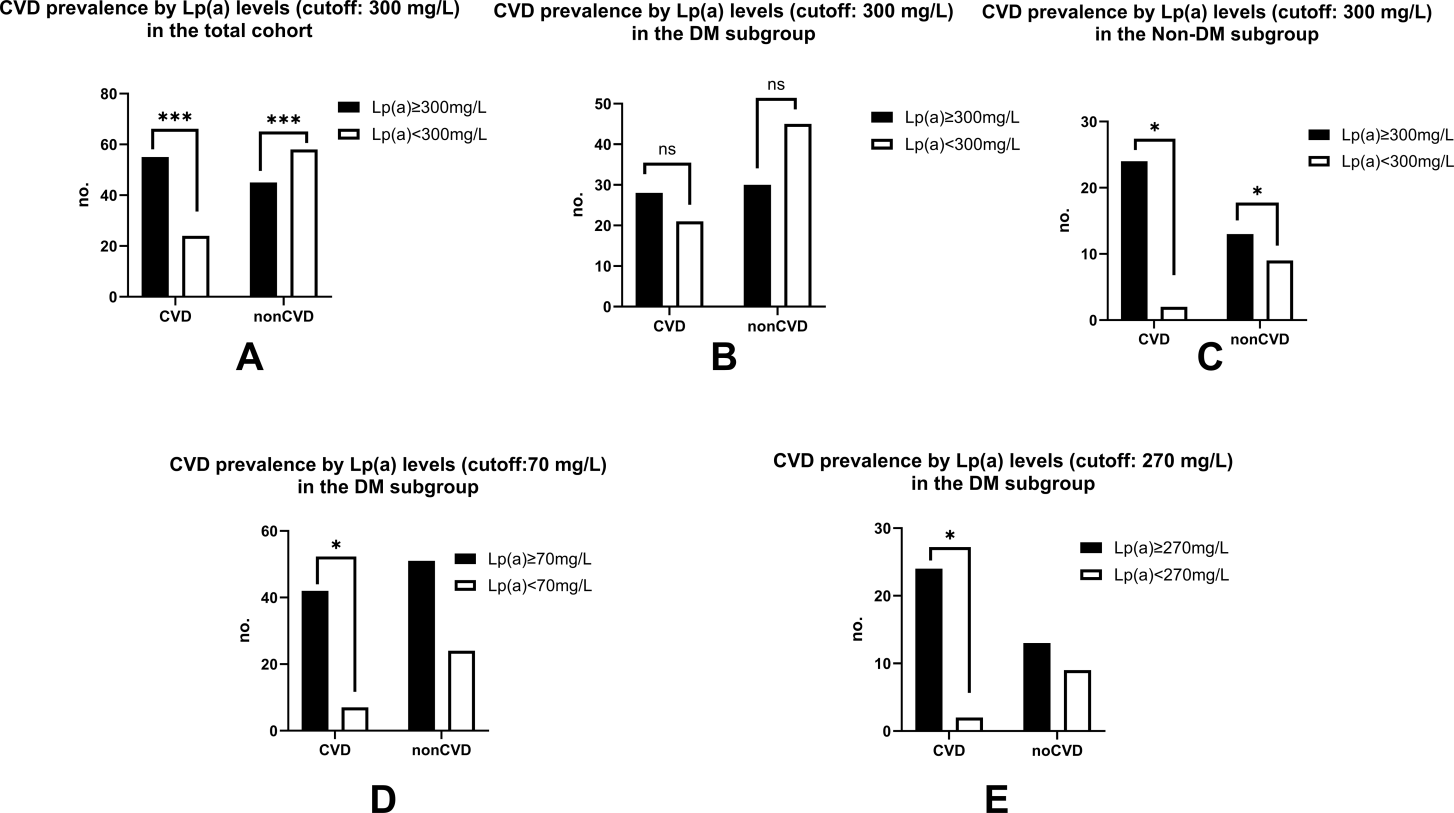
Association between stratified Lp(a) levels and CVD prevalence in different cohorts. **(A–C)** High-level thresholds: Comparison of CVD prevalence in individuals with Lp(a) > 300 mg/L versus those with lower levels in the general population **(A)**, patients with diabetes mellitus (DM) **(B)**, and non-DM individuals **(C)**. **(D, E)** Stratum-specific minimal thresholds: Identification of the minimum Lp(a) levels associated with a significant increase in CVD cases, specifically 70 mg/L for the DM group **(D)** and 270 mg/L for the non-DM group **(E)**. The *: p<0.05; ***: p<0.001; ns: no significance.

The results showed that, in the general group, the defined Lp(a) levels over reference 300mg/L was associated with an increased incidence of CVD and a reduced incidence of non-CVD than patients with Lpa<300. While this was consistent in the non-DM group, the increased cases observed in the DM group appeared to be insignificant. However, when the cutoff was adjusted to 70mg/L, the significance became obvious (p<0.05). In addition, it was also illuminating that the significant confined limitation was 270mg/L for non-DM, which was not as low as that for DM patients (p<0.05) (**Figure 1**) (**Table 2**).

**Table 2:** Antidiabetic use and statin use in DM patients with Lp(a) cutoff at 70mg/L.

History of drug use (n=126)	Lp(a)<70mg/L in DM	Lp(a)≥70mg/L in DM	P value
Statin (n=126)			0.541
Yes	15	51	
No	17	43	
Insulin (n=126)			0.072
Yes	27	63	
No	5	31	
SGLT-2i (n=126)			0.002**
Yes	22	33	
No	10	61	
GLP-1RA (n=126)			0.497
Yes	4	10	
No	28	84	
TZD (n=126)			0.328
Yes	3	2	
No	29	92	
Other OADs (n=125)			0.083
Yes	26	60	
No	6	33	

Lp(a), lipoprotein a; SGLT-2i, SGLT-2 inhibitors; GLP-1RA, glucagon-like peptide-1 receptor agonists; TZD, thiazolidinedione; OADs, oral antidiabetic drugs.The Bold values <0.05 stands for statistical significance. **: p<0.01.

Statistical Analysis: Comparisons between groups were performed using the Chi-square test or Fisher’s exact test.

### 3.2 Correlation between increased Lp(a) and the primary metabolic parameters in general, DM patients

We performed a Spearman correlation analysis to examine the relationship between increased Lp(a) levels and other primary metabolic parameters. The results revealed nuanced disparities between the two different groups.

**Figure 2:**
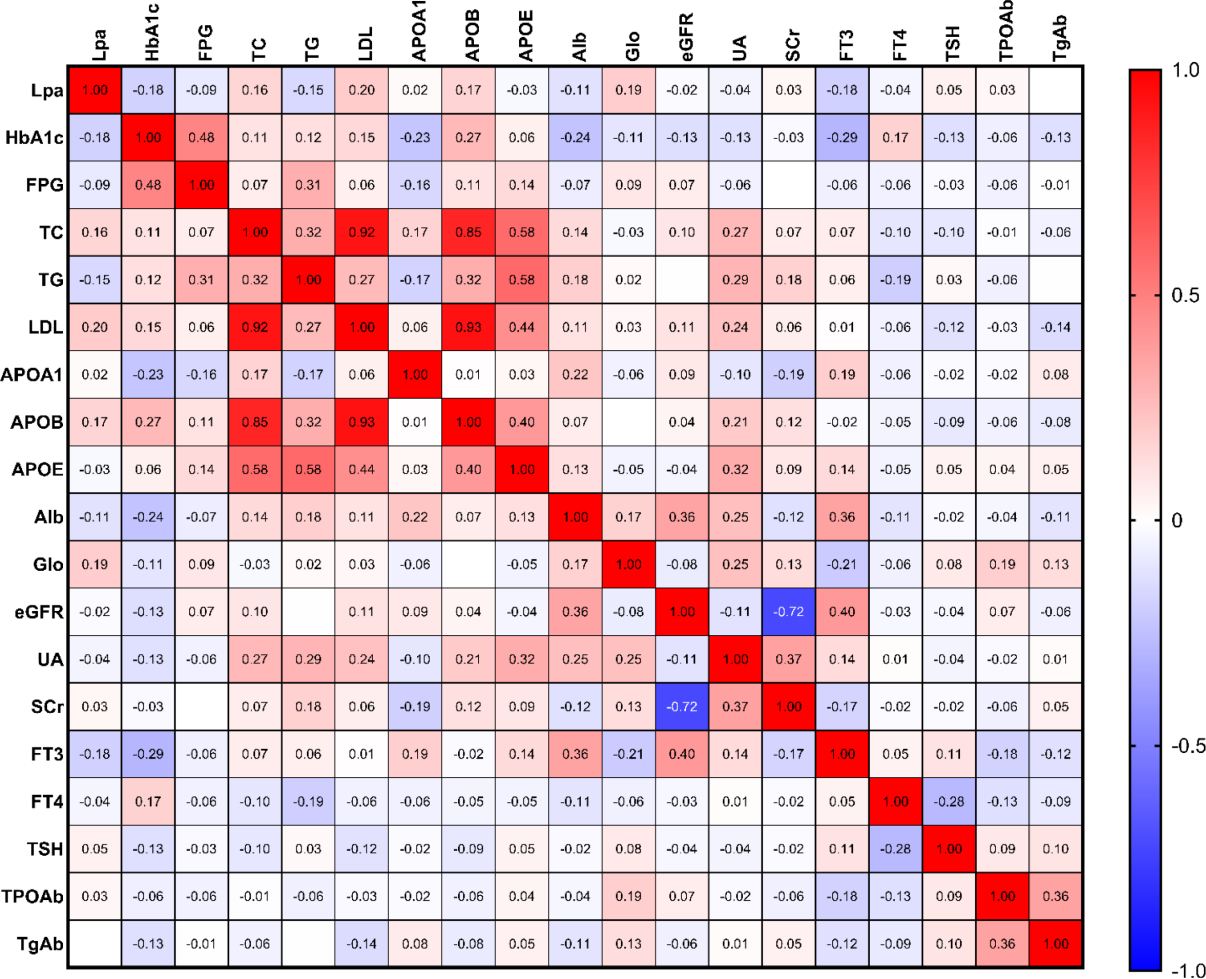
Heatmap analysis of Spearman’s correlation between Lp(a) levels and metabolic clinical parameters. This heatmap illustrates the strength and direction of the associations between Lp(a) levels and various metabolic factors in the general study population. Color Key: The color gradient represents the correlation coefficient (r), where red denotes a positive relationship, and blue represents a negative (inverse) relationship. The intensity of the color indicates the strength of the correlation. Key Associations: Notable inverse correlations (blue) were observed between Lp(a) levels and markers such as HbA1c and Triglycerides (TG). Statistical Significance: Correlation coefficients were calculated using Spearman’s rank correlation test to account for the non-normal distribution of Lp(a) levels. All metabolic parameters, including glucose control and lipid profiles (e.g., LDL-C and TG), were measured at baseline.

In the general group, the correlation analysis revealed that Lp(a) was positively correlated with total cholesterol (TC, r=0.158, p=0.034), low-density lipoprotein (LDL, r=0.204, p=0.006), apolipoprotein B (ApoB, r=0.168, p=0.03), Globin (r=0.195, p=0.009), but it was negatively correlated with glycated hemoglobin A1c (HbA1c,r=-0.179, p=0.021), triglycerides (TG, r=-0.151, p=0.043), and free triiodothyronine (FT3, r=-0.182, p=0.016) (**Figure 2**).

**Figure 3:**
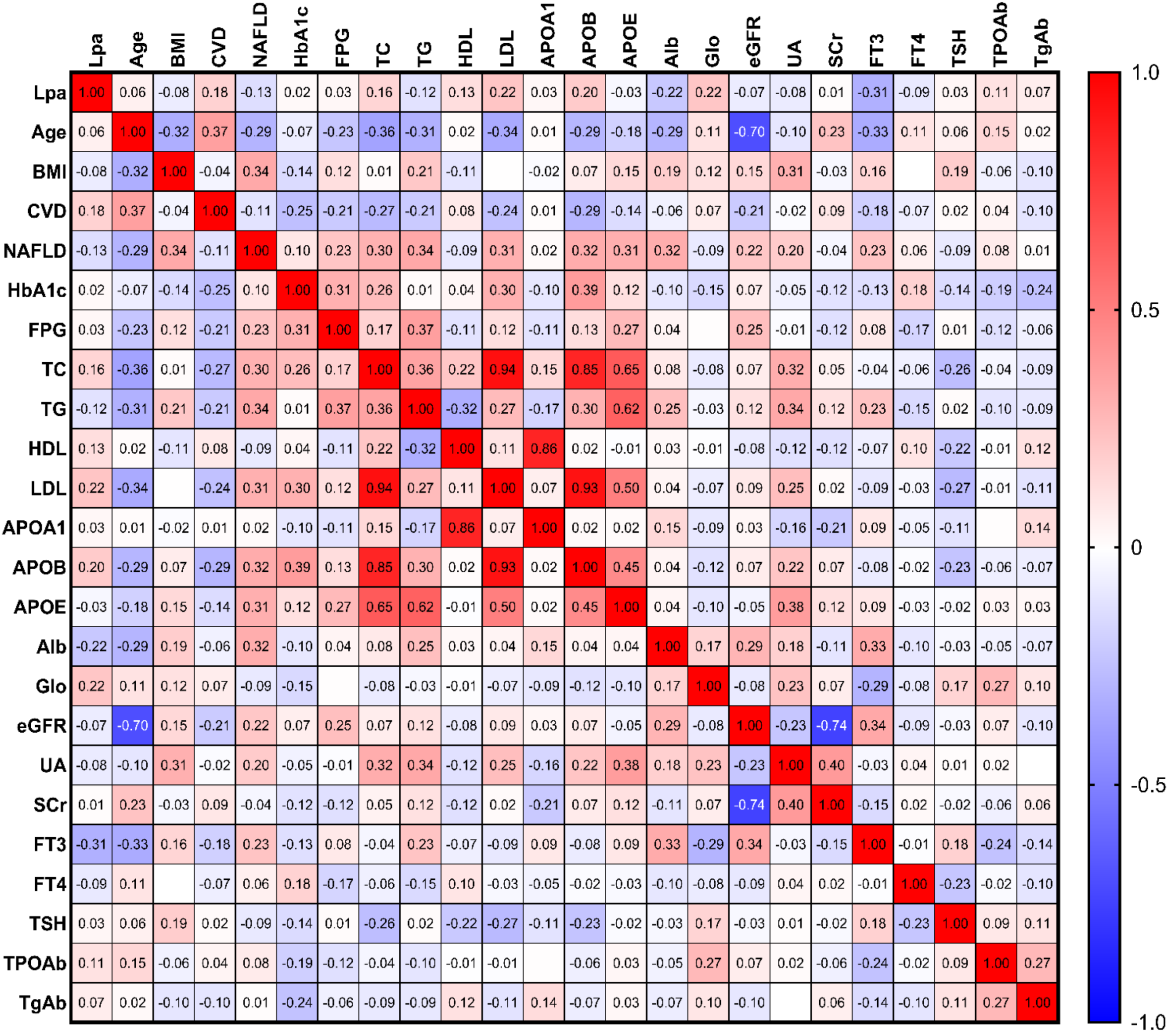
Heatmap of Spearman’s correlation coefficients between Lp(a) levels and metabolic parameters specifically in the DM group. This heatmap focuses on the diabetic cohort (n = 126) to illustrate the interrelationships between Lp(a) levels and clinical markers under diabetic conditions. Color Correlation: The color intensity represents the Spearman correlation coefficient (r), with red indicating a positive correlation and blue indicating a negative (inverse) correlation. Statistical Significance: r values were calculated using Spearman’s rank correlation to account for the skewed distribution of Lp(a).

In contrast to general patients, in the DM group an increase in Lp(a) levels was only positively associated with LDL(r=0.224, p=0.014), ApoB(r=0.202, p=0.035), and globin (r=0.222, p=0.015) but was negatively associated with albumin (Alb, r=-0.219, p=0.016) and FT3 (r=-0.311, p=0.0006) levels (**Figure 3**).

### 3.3 Stratified age groups revealed variability in the prevalence of non-alcoholic fatty liver disease and CVD in the general, DM, and DM groups with Lp(a)>70mg/L

**Figure 4:**
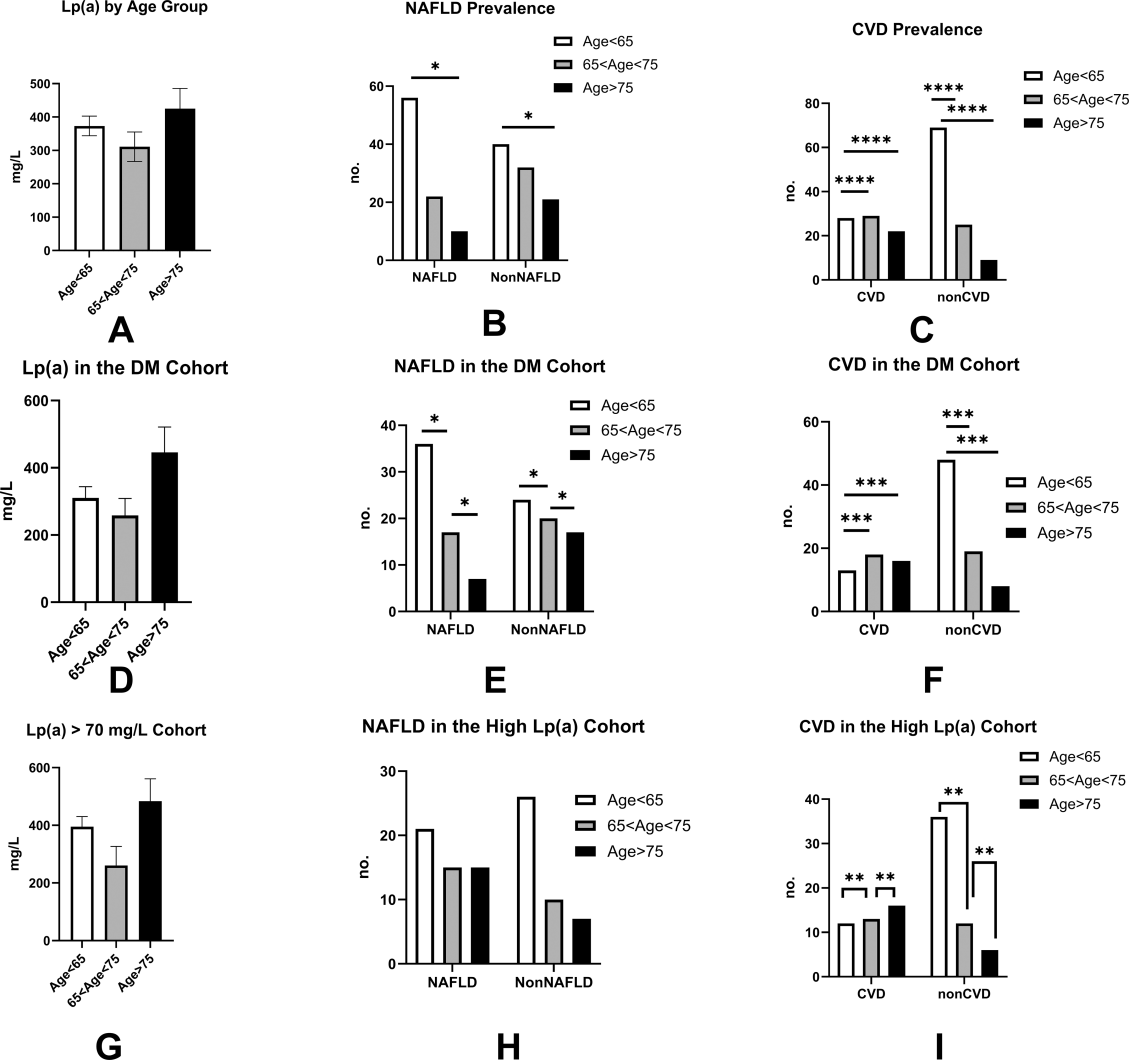
Stratified analysis of the associations between age, Lp(a) levels, and clinical comorbidities (NAFLD and CVD) . This figure illustrates how age differences correlate with Lp(a) levels and the prevalence of non-alcoholic fatty liver disease (NAFLD) and cardiovascular disease (CVD) in different clinical groups. **(A–I)** display data stratified by age: < 65 years (white bars), 65–75 years (gray bars), and > 75 years (black bars). **(A–C)** Total Cohort Analysis: **(A)** Distribution of serum Lp(a) levels across age groups; **(B)** Prevalence of NAFLD and Non-NAFLD; **(C)** Prevalence of CVD and Non-CVD; **(D–F)** Diabetes Mellitus (DM) Subgroup: **(D)** Serum Lp(a) levels within the DM cohort; **(E)** Prevalence of NAFLD stratified by age in DM patients; **(F)** Prevalence of CVD stratified by age in DM patients; **(G–I)** High Lp(a) Subgroup (Lp(a) > 70 mg/L): **(G)** Age distribution within the high Lp(a) cohort; **(H)** Prevalence of NAFLD in the high Lp(a) cohort; and **(I)** Prevalence of CVD in the high Lp(a) cohort. Lp(a) Stability with Age: Data indicate that variation in age has no significant effect on serum Lp(a) levels, suggesting that Lp(a) levels remain relatively stable across the lifespan in this cohort. Age and NAFLD Prevalence: In patients aged <75 years, age significantly influenced the prevalence of NAFLD in both the general population and the diabetes mellitus (DM) group. Notably, this age-related trend in NAFLD was not observed in the specific subgroup of DM patients with Lp(a)≥70 mg/L. Age and CVD Prevalence: Across all studied groups, the prevalence of CVD showed a significant association with the 65–75 years age bracket, highlighting this period as a critical window for prevalent cardiovascular complications. Statistical Methodology: Categorical variables were compared using the Chi-square test or Fisher’s exact test. *P < 0.05, **P < 0.01, ***P < 0.001, and ****P < 0.0001. Differences without symbols are not considered statistically significant (P > 0.05).

We continued to explore the significant implications of Lp(a) levels by age and employed a Chi-square test to reveal the effect of aging on the prevalence of NAFLD and CVD cases. Across all groups, Lp(a) did not display significant discrepancies with age variation (p=0.239). Although the NAFLD was increased in age<75 years (including age<65 and 65<age<75) patients in general and DM groups, the prevalence of CVD was profoundly in age between 65 to 75 years across all patients’ groups, and significantly higher in age>75 in Lp(a) >70mg/L of DM (**Figure 4**).

### 3.4 Correlation between Lp(a) levels and BMI, NAFLD, and CVD distribution across all age groups

We thereafter analyzed the relationship between Lp(a) levels and BMI, NAFLD, and CVD distributions. We found that in the general group, LP(a) levels were significantly associated with CVD in all age groups<75 years. At the same time, body mass index (BMI) was exclusively correlated with NAFLD (**Table 3**).

**Table 3:** Correlation between Lp(a) levels and BMI, NAFLD, and CVD distribution in the general group.

		Age<65	65<Age<75	Age>75
Variables	Correlations	CVD	CVD	CVD
Lp(a)	r	0.357	0.331	ns
	p	<0.0001	0.014	ns
		NAFLD	NAFLD	NAFLD
BMI	r	0.393	0.543	ns
	p	<0.0001	<0.0001	ns

Lp(a), lipoprotein a; BMI, body mass index; NAFLD, nonalcoholic fatty liver disease; CVD, cardiovascular diseases; ns, not significant.

Furthermore, the Lp(a) in the DM group was identified as positively related to increased CVD in subjects <65 years of age, and BMI was still associated with NAFLD in subjects between 65 and 75 years of age (**Table 4**).

**Table 4:** Correlation between Lp(a) levels and BMI, NAFLD, and CVD distribution in the DM group.

		Age<65	65<Age<75	Age>75
Variables	Correlations	CVD	CVD	CVD
Lp(a)	r	0.279	ns	ns
	p	<0.0001	ns	ns
		NAFLD	NAFLD	NAFLD
BMI	r	ns	0.508	ns
	p	ns	0.001	ns

Lp(a), lipoprotein a; BMI, body mass index; NAFLD, nonalcoholic fatty liver disease; CVD, cardiovascular diseases. ns, no significance.

### 3.5 Binary logistic regression analysis of Lp(a) and CVD in the DM group

We performed multivariable linear regression and binary logistic regression analyses to evaluate the association of Lp(a) levels and CVD prevalence within both the DM and non-DM cohorts. These analyses identified significant independent factors associated with the presence of CVD and variations in Lp(a) levels (**Tables 5**, **6**).

**Table 5:** Multivariable logistic regression analysis of factors associated with prevalent CVD in the DM group.

Model	-2 log likelihood	Nagelkerke R²						
DM	132.782	0.285						
Variable	B	SE	Wald	df	OR (95% CI)	BCa95%CI	P-value	Bootstrap p-value
Intercept	-3.070	1.610	3.638	1	0.046	-6.357~-0.360	0.056	0.058
HbA1c (%)	-0.224	0.102	4.836	1	**0.779 (0.654, 0.976)**	**-0.457~-0.073**	**0.028**	**0.024**
Age(Yrs)	0.067	0.020	11.710	1	**1.069 (1.029,1.111)**	**0.029~0.131**	**0.001**	**0.001**
Lp(a)≥70mg/L	0.919	0.516	3.173	1	2.508 (0.912-6.895)	-0.430~14.277	0.075	**0.045**
LDL-C (mmol/L)	-0.158	0.264	0.359	1	0.854 (0.509,1.431)	-0.794~0.416	0.549	0.596

HbA1c: glycated hemoglobin A1c; Alb: albumin; Lp(a): Lipoprotein a; LDL-C: low-density lipoprotein cholesterol. BCa: bias-corrected and accelerated. The dependent variable is the presence of CVD (coded as 1 = Yes, 0 = No). A binary logistic regression model with the “Enter” method was used to evaluate the independent discriminative value of Lp(a) ≥ 70 mg/L. Covariates, including Age, HbA1c, and LDL-C, were selected based on biological plausibility and the significant associations identified in the Spearman correlation heat map (**Figure 3**).The Bold values stands for risk ratio which is statistically significant.

**Table 6:** Multivariable linear regression analysis identifying independent metabolic factors associated with serum Lp(a) levels in the DM group (n = 126).

Model summary	R	R^2^	Adjusted R^2^	P-value	Colinearity	
	0.298	0.089	0.074			
Variables	**Unstandardized B**	**SE**	**t**	**Sig**	**Tolerance**	**VIF**
Constant	1148.156	240.939	4.765	<0.0001		
FT3(pmol/L)	-82.471	38.265	-2.155	0.033	0.928	1.077
Alb(g/L)	-10.431	5.080	-2.053	0.042	0.928	1.077

FT3, free triiodothyronine; Alb, albumin; TPOAb, anti-Thyroid peroxide autoimmune antibody; Glo, globin. This linear regression model explores the independent biological determinants of Lp(a) levels within the diabetic population (n = 126). The dependent variable is the serum Lp(a) level (mg/L). The independent variables are FT3 (free triiodothyronine) and Alb (albumin). Multicollinearity was strictly assessed using Variance Inflation Factors (VIF). All VIF values were < 1.1, and Tolerance values were > 0.9, indicating that the associations for FT3 and Alb are statistically independent and free from collinearity bias. The standardized coefficients (β) indicate that lower levels of FT3 and Alb are independently associated with higher Lp(a) levels, suggesting a potential metabolic regulation of Lp(a) synthesis or clearance in diabetes. R = 0.298; Adjusted R^2^ = 0.074. While Lp(a) is primarily genetically determined, these metabolic factors significantly contribute to its phenotypic variation in this clinical setting.The bold value stands for statistically significant.

All Odds Ratios (ORs), 95% Confidence Intervals (CIs), and P-values were derived from 1,000 bias-corrected and accelerated (BCa) bootstrap resamples to ensure robust estimates in this retrospective cohort. The model demonstrated adequate calibration (Hosmer-Lemeshow test, P > 0.05) and explanatory power, as indicated by a Nagelkerke R^2^ of 0.285.

### 3.6 Discriminative performance of elevated Lp(a) levels for CVD outcomes in DM and non-DM patients as evaluated by ROC curves

**Figure 5:**
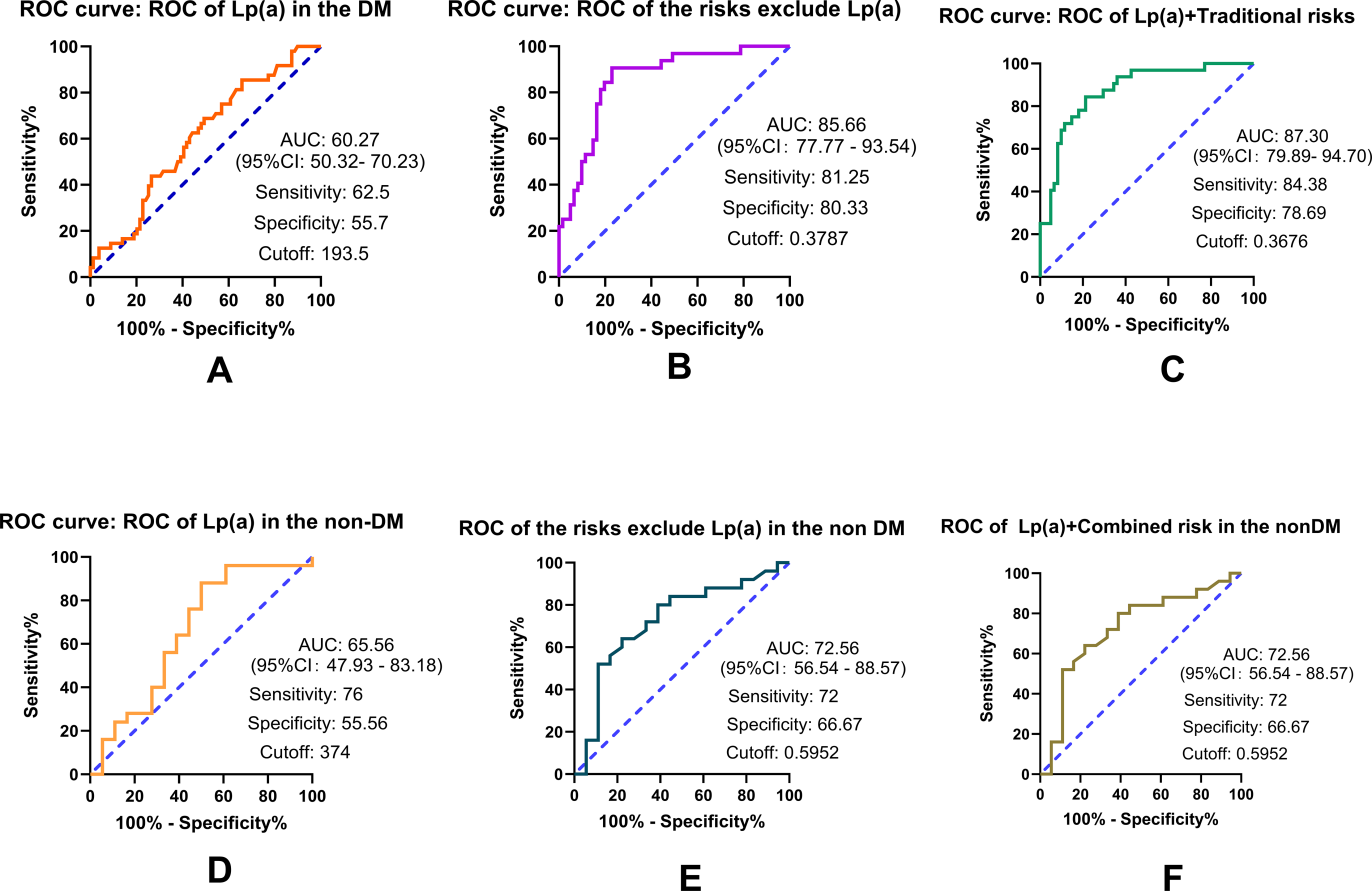
ROC curve analysis demonstrating the discriminative performance of Lp(a) and integrated clinical models for prevalent CVD. This figure evaluates the diagnostic accuracy of Lp(a) and multivariable models in identifying cardiovascular disease (CVD) within the study cohort. Individual Lp(a) Performance: **(A)** and **(D)** illustrate the discriminative utility of Lp(a) alone in patients with diabetes mellitus (DM) and non-DM individuals, respectively. The area under the curve (AUC), along with the sensitivity, specificity, and optimal cutoff values, was determined using the Youden index. Baseline vs. Integrated Models: **(B)** and **(E)** represent the performance of baseline models (comprising age, HbA1c, and LDL-C, but excluding Lp(a)). **(C)** and **(F)** display the integrated models, in which Lp(a) was combined with baseline parameters via binary logistic regression. Statistical Validation: To ensure the stability of the discriminative performance, all AUCs and 95% confidence intervals (CIs) were validated using 1,000-sample bias-corrected and accelerated (BCa) bootstrapping. Model Goodness-of-Fit: The integrated models showed superior discriminative ability compared to the baseline models, and their fit was confirmed by the Hosmer-Lemeshow test (P > 0.05).

We performed a ROC analysis to examine the discriminative utility of Lp(a) in DM and non-DM patients. The results revealed that Lp(a) showed moderate discriminatory ability for CVD. When integrated with traditional parameters identified in regression analyses, the addition of Lp(a) to the baseline model resulted in a numerical increase in the AUC from 0.86 (95% CI: 0.78–0.93) to 0.87 (95% CI: 0.80–0.95) in the DM group. Although the magnitude of the increment in the AUC was modest, it suggests a potential complementary role for Lp(a) in cardiovascular risk stratification. In contrast, while Lp(a) also functioned as a sensitive marker for CVD in non-DM patients, its addition to the traditional model did not significantly improve the AUC for CVD outcomes in this cohort (**Figure 5**).

## 4 Discussion

The present study revealed a critical, previously underappreciated interaction between Lp(a) levels and Diabetes Mellitus (DM) that necessitates a profound reassessment of clinical stratification for prevalent CVD. The central, most striking finding was that the optimal cutoff of Lp(a) for identifying CVD status is markedly lower in DM patients compared to non-diabetic subjects. Furthermore, the study highlighted the enhanced, context-specific discriminative utility of Lp(a) in the DM milieu, demonstrating that the addition of Lp(a) significantly increases the AUC for CVD identification within the DM cohort. These results strongly support the hypothesis that Lp(a) serves as a potent independent factor associated with residual cardiovascular risk in DM, persisting even after adjusting for HbA1c, LDL-C, and age.

The most crucial finding, was the marked discrepancy in the Lp(a) threshold associated with CVD presence between the DM and non-DM cohorts. Specifically, the optimal cutoff level of Lp(a) for discriminating CVD status was identified as 70 mg/L in patients with DM —a threshold approximately fourfold lower than the 270 mg/L observed in non-DM subjects. These findings suggest a stronger association between low-level Lp(a) elevations and cardiovascular outcomes within the diabetic milieu. These findings also advocate for a re-evaluation of current clinical stratification strategies, suggesting that DM patients may benefit from more stringent, individualized Lp(a) thresholds to facilitate the more precise identification of those with a high cardiovascular risk.

A notable and seemingly counterintuitive finding in our study was the significant negative correlation between Lp(a) levels and metabolic markers, such as HbA1c and TG, within the DM cohort. While all lipid-related risk factors are traditionally expected to trend together, this inverse relationship has been documented in several large-scale prospective studies (21–23). Biologically, this may be attributed to the inhibitory effect of insulin on apo(a) gene expression in hepatocytes; as HbA1c levels rise, the compensatory hyperinsulinemia typically seen in T2DM may paradoxically suppress Lp(a) synthesis (24, 25). Furthermore, we considered the potential for statistical artifacts arising from therapeutic interventions. The widespread use of statins in the DM group—which effectively lowers TG but is known to modestly increase Lp(a)—could partially account for this decoupled relationship (26). To minimize confounding factors, we adjusted our models for nutritional status (via serum Alb) and thyroid function (FT3), both of which are key determinants of protein synthesis and lipid metabolism. The persistence of Lp(a) as a significant discriminator of CVD prevalence, despite these inverse correlations, reinforced its role as a unique, independent factor associated with residual cardiovascular burden. This suggests that Lp(a) operates through distinct pathological pathways within the diabetic milieu, independently of glucose and lipid metabolism.

Our analysis of correlational relationships provided crucial context: Lp(a) was significantly correlated with classic atherogenic markers, such as LDL and ApoB, in both the general and DM cohorts. However, the discovery of negative correlations between Lp(a) levels and key metabolic markers, including HbA1c and TG, highlighted the complex and non-linear nature of the relationship between Lp(a) and established factors, given their documented role in CVD. The stability of Lp(a) levels across different age groups reinforced its genetic determination. Meanwhile, the higher prevalence of CVD observed in the 65–75 age group contextualized the associated clinical burden and was found to be consistent with previous lifelong investigations into the long-term association between Lp(a) levels and cardiovascular outcomes in female populations (27).

Logistic regression further highlighted age as a significant independent CVD predictor in the DM group, and the negative HbA1c coefficient suggested that in the DM group, the HbA1c should be targeted according to individual characteristics, such as age (28). The ROC curve analysis provided compelling evidence of Lp(a)’s clinical utility. The fact that Lp(a) significantly enhanced the AUC for CVD identification exclusively in the DM cohort confirmed that Lp(a) captures essential information regarding the residual cardiovascular burden specific to the diabetic milieu (29). It should also be noted that the AUC for Lp(a) in predicting CVD was found to be 0.60 (95% CI: 0.50–0.70), indicating a moderate level of discrimination. This suggests that while Lp(a) is independently associated with CVD risk in diabetic patients, it should be integrated with other clinical parameters rather than used as a standalone predictor for individual risk assessment.

This synergistic enhancement strongly suggests that Lp(a)’s behavior is governed by qualitative molecular modification rather than simple concentration. Our hypothesis centers on the non-enzymatic glycation of Lp(a) in a chronic hyperglycemic environment, a concept supported by a growing body of basic research (30–32). The Lp(a) measured at 70 mg/L in a DM patient is therefore a fundamentally more potent atherogenic molecule than the same concentration in a non-DM individual, which is the biological driver of both the lower clinical threshold and the superior incremental AUC observed in our models.

In this study, we observed a discrepancy between the optimal diagnostic cutoff derived from ROC analysis (19 mg/dL) and the risk threshold identified via iterative threshold evaluation (7 mg/dL). This divergence is statistically and clinically meaningful. The ROC-derived cutoff point represents the point of maximum simultaneous sensitivity and specificity for differentiating CVD status. In contrast, the 7 mg/dL threshold identified through iterative evaluation represents the ‘minimal risk inflection point,’ suggesting that, for patients with DM, the pathological contribution of Lp(a) may begin at much lower concentrations than previously recognized in general populations. This finding reinforces our central hypothesis that the cardiovascular risk associated with Lp(a) in a diabetic background may lack a truly ‘safe’ baseline.

This robust mechanistic rationale has immediate implications for global clinical practice and the deployment of novel therapies. Current guidelines recommend screening for Lp(a) at least once to identify patients at high cardiovascular risk (33). Our data argue forcefully that DM patients should be subject to a stricter, lower action threshold than the general population. Antisense Oligonucleotides (ASOs) and Small interfering RNAs (siRNAs) have demonstrated the potential for far greater Lp(a) reductions (34). Ongoing clinical outcomes trials (CVOTs) are essential for confirming the clinical benefit of PCSK9 in reducing Lp(a) (35). Our findings suggest that DM patients with Lp(a) levels between 70 mg/L and 270 mg/L should be prioritized for intervention and inclusion in these trials, as their Lp(a) is functionally amplified, making them an ideal high-risk group to maximize the observed therapeutic benefit.

### 4.1 Limitations

The primary limitations of this study prevent the establishment of causality for future CVD events. However, this is a primary trial that evaluated the efficacy of Lp(a) for CVD risk in DM patients, along with its sensitivity and specificity in managing CVD risk in patients with DM. While our study identified significant factors associated with CVD, the relatively small sample size may result in a lower Events-Per-Variable (EPV) ratio for some models. This introduces the potential for model overfitting, whereby the discriminative performance may appear overly optimistic for this specific cohort. Although we addressed this issue through internal validation via bootstrapping to adjust for optimism, the results should be interpreted with caution. Larger, multi-center prospective studies are required to confirm the generalizability of the proposed Lp(a) discriminative thresholds and to establish their prospective predictive value.

## 5 Conclusion

In summary, our study identified Lp(a)≥70 mg/L as a significant factor associated with the prevalence of CVD in patients with diabetes, independent of glycemic control and traditional lipid markers. However, given the retrospective and cross-sectional design of this article, these findings should be considered hypothesis-generating. Future prospective longitudinal studies are warranted to validate whether this threshold can effectively predict the incidence of future cardiovascular events and to explore the potential benefits of targeted Lp(a)-lowering therapies for this specific population.

## Data Availability

The data that support the findings of this study are not publicly available due to privacy reasons but are available from the corresponding author upon request.
